# Supplementary material for: Acidified Nitrite Accelerates Wound Healing in Type 2 Diabetic Male Rats: A Histological and Stereological Evaluation
Source: Molecules. 2021 Mar 26;26(7):1872. doi: 10.3390/molecules26071872 (PMC8037216; doi:10.3390/molecules26071872)
Supplement: Supplementary file 1 [file molecules-26-01872-s001.pdf]

molecules

MDPI

Supplementary Materials

Acidified Nitrite Accelerates Wound Healing in Type 2 Diabetic Male Rats: A Histological and Stereological Evaluation

Hamideh Afzali [1,2], Mohammad Khaksari [1], Sajad Jeddi [2], Khosrow Kashfi [3], Mohammad-Amin Abdollahifar [4,*] and Asghar Ghasemi [2,*]

[1] Endocrinology and Metabolism Research, and Physiology Research Centers, Kerman University of Medical Sciences, Kerman, 7616913555, Iran; hamide_afzali@yahoo.com (H.A.); mkhaksari@kmu.ac.ir (M.K.).

[2] Endocrine Physiology Research Center, Research Institute for Endocrine Sciences, Shahid Beheshti University of Medical Sciences, Tehran, 1985717413, Iran; Sajad.jeddi@sbmu.ac.ir

[3] Department of Molecular, Cellular and Biomedical Sciences, Sophie Davis School of Biomedical Education, City University of New York School of Medicine, New York, NY 10031, USA; Kashfi@med.cuny.edu

[4] Department of Biology and Anatomical Sciences, Faculty of Medicine, Shahid Beheshti University of Medical Sciences, Tehran, 1985717413, Iran

* Correspondence: abdollahima@sbmu.ac.ir (M.-A.A.); Tel.: +982123872555; Ghasemi@endocrine.ac.ir (A.G.); Tel.: +982122432489

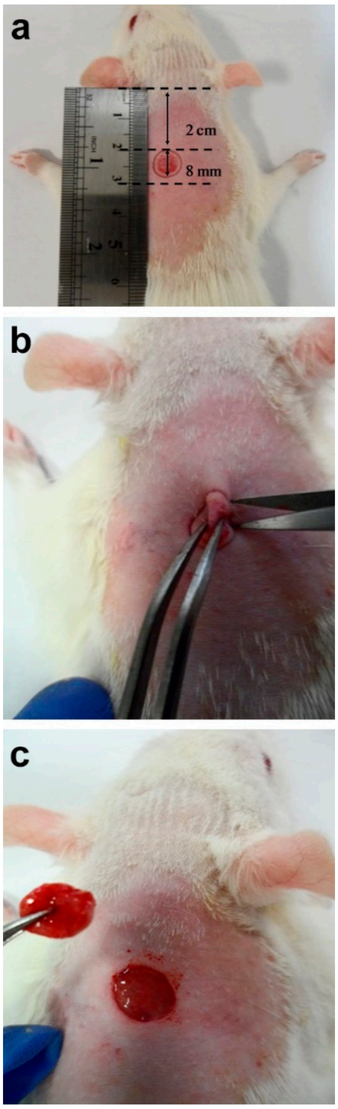

Figure S1. Wound induction. An 8 mm punch is used to mark the location of the excisional wound (2 cm below the ears) (a). Forceps retract the skin outward and scissors are used to sharply excise a circular piece of skin down through the panniculus carnosus (b). One full-thickness excisional wound (c).
